# CASCADING BURDEN OF UNCONTROLLED CHRONIC CONDITIONS AMONG PERSONS LIVING WITH DEMENTIA

**DOI:** 10.1093/geroni/igae098.2177

**Published:** 2024-12-31

**Authors:** Rachel O’Conor, Allison Pack, Andrea Russell, Dianne Oladejo, Darby Morhardt, Lee Lindquist, Michael Wolf

**Affiliations:** Northwestern University, Evanston, Illinois, United States; Northwestern University, Evanston, Illinois, United States; Northwestern University, Evanston, Illinois, United States; Northwestern University, Evanston, Illinois, United States; Northwestern University, Evanston, Illinois, United States; Northwestern University, Evanston, Illinois, United States; Northwestern University, Evanston, Illinois, United States

## Abstract

Half of all potentially avoidable hospitalizations among persons living with dementia (PLWD) are due to poorly controlled chronic conditions. Yet little is known about how PLWD and their caregivers manage the multiple chronic conditions (MCC) and polypharmacy they often contend with, or how these health management behaviors impact PLWD and caregiver well-being. We conducted qualitative interviews with patient-caregiver dyads exploring capacity to manage MCC. PLWD were eligible if they had mild or moderate dementia, ≥3 chronic conditions, ≥5 medicines, and a caregiver. Three coders applied a priori and emergent codes to transcripts. We interviewed 68 participants (PLWD: mean age 78 years, 65% female, 53% white, 32% Black; Caregivers: mean age 65 years, 73% female, 56% spouse, 38% adult child). PLWD were managing 5 chronic conditions and took 9 medicines on average. PLWD recalled instances of difficulty managing MCC and subsequent hospitalization, while emphasizing they had recovered. Following errors in health management behaviors, they expressed gratitude for additional caregiver support. In contrast, caregivers described a cascading burden of uncontrolled chronic conditions among PLWD. Many reported feeling overwhelmed managing the complexity of MCC, including multi-drug regimens, for the PLWD. Caregivers cited difficulties achieving adequate control for numerous chronic conditions (e.g. hypertension, diabetes, heart disease). Consequences included hospitalizations and increased regimen complexity; in turn, these increased caregiver responsibilities, emotional strain, and financial costs. Findings suggest management of MCC compounds caregiving responsibilities and contributes to caregiving burden. Supporting caregivers’ practical skills for managing MCC may promote PLWD and caregiver well-being.

